# Roles of Selenoproteins in Brain Function and the Potential Mechanism of Selenium in Alzheimer’s Disease

**DOI:** 10.3389/fnins.2021.646518

**Published:** 2021-03-08

**Authors:** Zhong-Hao Zhang, Guo-Li Song

**Affiliations:** ^1^Shenzhen Key Laboratory of Marine Bioresources and Ecology, College of Life Sciences and Oceanography, Shenzhen University, Shenzhen, China; ^2^Shenzhen Bay Laboratory, Shenzhen, China; ^3^Shenzhen-Hong Kong Institute of Brain Science-Shenzhen Fundamental Research Institutions, Shenzhen, China

**Keywords:** selenoprotein, neurotransmission, brain function, Alzheimer’s disease, Ca^2+^ homeostasis

## Abstract

Selenium (Se) and its compounds have been reported to have great potential in the prevention and treatment of Alzheimer’s disease (AD). However, little is known about the functional mechanism of Se in these processes, limiting its further clinical application. Se exerts its biological functions mainly through selenoproteins, which play vital roles in maintaining optimal brain function. Therefore, selenoproteins, especially brain function-associated selenoproteins, may be involved in the pathogenesis of AD. Here, we analyze the expression and distribution of 25 selenoproteins in the brain and summarize the relationships between selenoproteins and brain function by reviewing recent literature and information contained in relevant databases to identify selenoproteins (GPX4, SELENOP, SELENOK, SELENOT, GPX1, SELENOM, SELENOS, and SELENOW) that are highly expressed specifically in AD-related brain regions and closely associated with brain function. Finally, the potential functions of these selenoproteins in AD are discussed, for example, the function of GPX4 in ferroptosis and the effects of the endoplasmic reticulum (ER)-resident protein SELENOK on Ca^2+^ homeostasis and receptor-mediated synaptic functions. This review discusses selenoproteins that are closely associated with brain function and the relevant pathways of their involvement in AD pathology to provide new directions for research on the mechanism of Se in AD.

## Introduction

Over the past decades, selenium (Se) and its compounds have mainly been the focus of research on regulation of development and the immune system and on antitumor properties due to their strong antioxidant activities (Rayman, 2000; Schomburg, 2016). Nutritional data show that under normal diet conditions, the Se level is highest in the kidney, followed by the liver, spleen, pancreas, heart, and brain (Chen and Berry, 2003). However, when Se uptake is insufficient, this ranking changes according to the priority order of different organs for Se. Among organs, the brain retains Se the longest (Burk et al., 1972; Brown and Burk, 1973), indicating the importance of Se in the maintenance of physiological function in the central nervous system (CNS). Epidemiological surveys show a significant positive correlation between the Se level and cognitive ability—i.e., a dose-response effect (Gao et al., 2007)—and that the blood Se level gradually decreases with age (Berr et al., 1993). Furthermore, Se levels change significantly in the brain and blood of patients with various neurodegenerative diseases [such as Alzheimer’s disease (AD), Parkinson’s disease (PD), multiple sclerosis, and Batten’s disease] (Chen and Berry, 2003). Therefore, the role of Se in the brain and in neurodegenerative diseases has gradually become a research hotspot.

AD is an age-related neurodegenerative disease with a very high prevalence among elderly people. The Se level in AD patients and carriers of the apolipoprotein E (ApoE4) allele (a high risk factor for sporadic AD) is significantly decreased (Cardoso et al., 2010), suggesting that Se deficiency is associated with AD. Senile plaques generated by β-amyloid (Aβ) deposition and neurofibrillary tangles (NFTs) formed by tau hyperphosphorylation in the brain are two major pathological features of AD. Early studies showed that Se supplementation in SH-SY5Y cells expressing a mutant amyloid precursor protein (APP) gene reduced lipid peroxidation product levels, inhibited β-secretase 1 (BACE1), and γ-secretase activity, and reduced Aβ aggregation (Gwon et al., 2010). Sodium selenate, an inorganic Se compound, was once considered a useless Se compound because of its low bioavailability (Daniels, 1996). Interestingly, van Eersel et al. (2010) and Jin et al. (2017) used transgenic animal models of AD to show that sodium selenate reduced tau protein phosphorylation and ameliorated cognitive impairment in AD mice by regulating the activity of protein phosphatase 2A (PP2A) (Ishrat et al., 2009; Lovell et al., 2009; Corcoran et al., 2010; van Eersel et al., 2010; Jin et al., 2017), which undoubtedly promoted research regarding Se in AD prevention and treatment. Organic Se has attracted attention due to its advantages, including its enhanced biological activity, decreased toxicity, and diminished environmental pollution concern compared to inorganic Se. Many organic Se compounds have been shown to have therapeutic effects in AD mice models (Xie et al., 2017, 2018; Zhang et al., 2017a, 2018). Through a series of studies, Zhang et al. showed that selenomethionine enhanced the antioxidant capacity, mitigated Aβ and tau pathology, reversed synaptic deficits, and ameliorated cognitive decline in AD mice (Song et al., 2014; Zhang et al., 2016, 2017a, 2018), demonstrating the multitarget effect of Se in AD treatment. Furthermore, impaired autophagy (Zhang et al., 2017b,c; Song et al., 2018) and mitochondrial dysfunction (Balaban et al., 2017) have been shown to be the targets of Se compounds in AD. However, the in-depth mechanisms of Se in AD prevention and treatment remain unclear, and this lack of knowledge is a major factor limiting further clinical application of Se in AD and a reasonable explanation for the unsuccessful randomized clinical trial of Se in the AD study of Kryscio et al. (2017), which was based on the Se and vitamin E Cancer Prevention Trial.

Though there are several Se utilization pathways in the body, selenoprotein synthesis is the main means by which Se exerts numerous biological functions. Currently, genes encoding 25 selenoproteins have been identified in human genomic sequences (Kryukov et al., 2003). Glutathione peroxidase (GPX) was the first identified selenoprotein and participates in redox reactions. Se-containing GPX isozymes (GPX1, GPX2, GPX3, GPX4, and GPX6) exhibit different tissue-specific expression patterns and subcellular localization (Brigelius-Flohe, 1999). Thioredoxin reductases (TrxRs) (TXNRD1, TXNRD2, and TXNRD3) are other antioxidases that contribute not only to the antioxidant system but also to cell proliferation and apoptosis (Mustacich and Powis, 2000). Thyroid hormone deiodinases (DIOs) (DIO1, DIO2, and DIO3) participate in T3 and T4 production and regulate thyroid hormone activities (Kohrle, 2000). Methionine-sulfoxide-reductase 1 (MSRB1), also called SELENOR, is responsible mainly for repairing methionine-oxidized proteins. Interestingly, selenophosphate synthetase 2 (SEPHS2) (an enzyme involved in selenocysteine (Sec) biosynthesis) is also a selenoprotein. As a plasma Se transport protein, SELENOP also exhibits lipid hydroperoxide reductase activity (Saito, 2020). SELENOK, SELENOM, SELENOF, SELENOS, SELENOT, DIO2, and SELENON are endoplasmic reticulum (ER)-resident selenoproteins and participate mainly in the regulation of physiological processes, including Ca^2+^ flux, protein folding, and ER stress (Pitts and Hoffmann, 2018). SELENOO, the largest mammalian selenoprotein, possesses a protein kinase-like domain and may have a function in oxidative stress response (Lenart and Pawlowski, 2013). Sporadic reports have addressed other selenoproteins (SELENOW, SELENOH, SELENOI, SELENOU, and SELENOV), which still lack clear recognition except for their antioxidant function.

Biochemical and bioinformatic analyses have shown that most selenoproteins are expressed in the brain (Zhang et al., 2008) and that some selenoproteins are closely associated with brain function. The tRNA[Ser]Sec (*Trsp*) gene is required for the expression of all functional selenoproteins. Neuron-specific *Trsp*-knockout mice had significantly decreased expression levels of selenoproteins in the brain and showed delayed growth, loss of balance, and extensive neuronal degeneration (Wirth et al., 2010). Additionally, Selenop^–/–^ mice exhibited many features of neurological dysfunction (Hill et al., 2004; Schweizer et al., 2005). GPX4-regulated ferroptosis can induce progressive cognitive impairment and hippocampal neurodegeneration in mice (Hambright et al., 2017). Thus, these brain function-related selenoproteins may be involved in the occurrence and development of AD. However, the selenoproteins involved and their roles in these processes remain unclear. This review analyzes the expression and distribution of selenoproteins in the brain, assesses the associations between various selenoproteins and brain function and the potential of these selenoproteins in AD research, and discusses the roles of these selenoproteins in AD pathology.

### Expression and Distribution of Selenoproteins in the Brain

**FIGURE 1:**
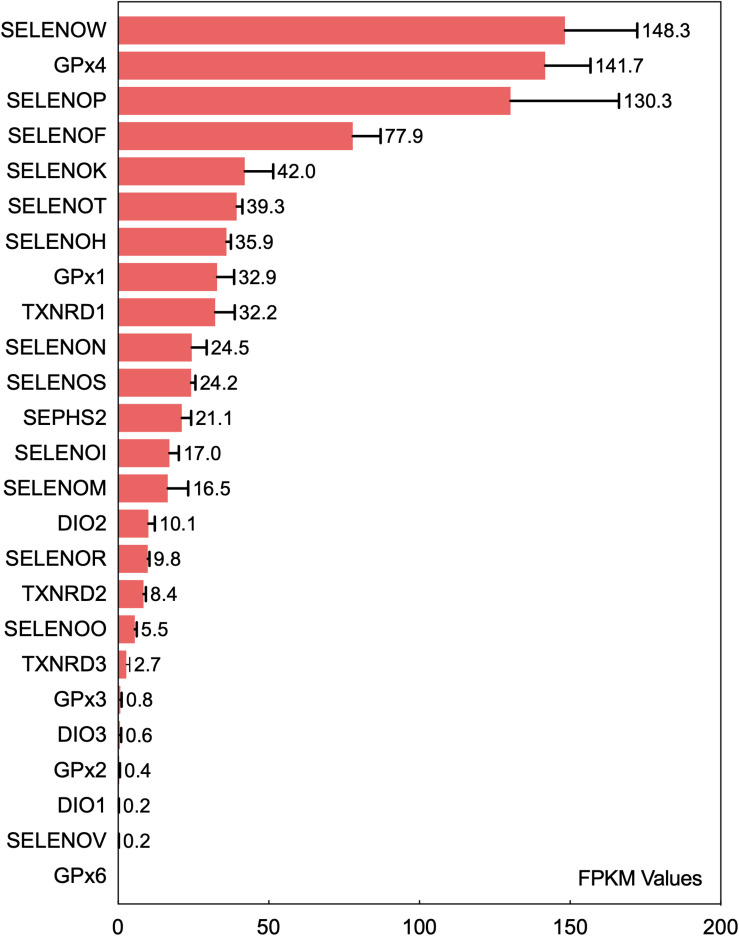
Ranking of the mRNA expression levels of 25 selenoproteins in the human brain. The fragments per kilobase of exon model per million mapped reads (FPKM) values of 25 selenoproteins in the brain obtained from gene transcriptome data were analyzed to rank selenoproteins based on their expression level in the human brain. The data are from the literature (Fagerberg et al., 2014).

Insertion of UGA-encoded Sec into a translating selenoprotein polypeptide is a complex and sophisticated protein translation process controlled synergistically by multiple regulatory factors (Bulteau and Chavatte, 2015). Differential expression levels of selenoproteins in tissues and organs are directly associated with the biological functions of selenoproteins. The current data regarding the differential expression levels of various selenoproteins in the brain originated from the bioinformatic studies conducted by Zhang et al. (2008). The transcripts of 24 selenoproteins are expressed in the mouse brain. The expression levels of GPX4, SELENOK, SELENOM, SELENOW, and SELENOF in the brain are higher than those of other selenoproteins, and GPX4, SELENOP, and SELENOW are expressed at high levels in more than 90% of brain regions. A recent gene transcriptomic analysis (Fagerberg et al., 2014) ranked the expression levels of 25 selenoproteins in the human brain (Figure 1). Among all selenoproteins, 6 (GPX3, DIO3, GPX2, DIO1, SELENOV, and GPX6) were confirmed to have very low or no expression in the human brain. SELENOW, GPX4, SELENOP, SELENOF, and SELENOK have the five highest expression levels, and this pattern is basically consistent with that observed in the mouse brain (Zhang et al., 2008). The next most highly expressed selenoproteins, in decreasing order, are SELENOT, SELENOH, GPX1, TXNRD1, SELENON, SELENOS, SEPHS2, SELENOI, and SELENOM. Six ER-resident selenoproteins (except for DIO2), especially SELENOF, SELENOK, and SELENOT, are expressed at relatively high levels in the brain. In addition, the selenoenzymes GPX4, GPX1, TXNRD1, and SEPHS2 are expressed at high levels in the brain. SELENOP is highly expressed in the brain due to its Se transport function. Among all selenoproteins, SELENOW exhibits the highest expression level in the brain; however, this expression profile is not consistent with the current understanding of its biological functions in the brain. SELENOI is another selenoprotein that has only recently begun to be studied and may perform certain physiological functions because of its expression in the brain.

Furthermore, differences in the spatial expression levels of selenoproteins also determine their physiological functions in the brain (Zhang et al., 2008). The spatial expression profile data of selenoproteins in the human brain are incomplete. Zhang et al. (2008) thoroughly analyzed *in situ* hybridization data and the expression profiles of more than 210,000 genes in a genome-wide expression database of the brain of adult mice in the previously published Allen Brain Atlas (ABA) and showed that brain selenoproteins are enriched mainly in the hippocampus, olfactory bulb, neocortex, and cerebellar cortex; the expression levels of GPX4, SELENOW, and SELENOF are highest in these four regions (Zhang et al., 2008). Except for SELENOP, all selenoprotein genes with high expression levels in the brain have at least moderate expression levels in the hippocampal region. The oculomotor nucleus, Edinger-Westphal nucleus, nucleus raphe pontis, anteroventral periventricular nucleus, and dorsal premammillary nucleus have the lowest expression levels of selenoproteins, indicating that these regions might be less dependent on selenoproteins and Se (Zhang et al., 2008).

### Selenoproteins and Brain Function

#### Brain and Neural Development

The importance of Se in brain development was confirmed in Selenop^–/–^ mice (Schomburg et al., 2003). In a low-Se environment, newborn Selenop^–/–^ mice exhibit severe hypoplasia and death (Hill et al., 2004; Schweizer et al., 2005). However, after overexpression of SELENOP in hepatocytes, the above neuropathological features were reversed when the mice were fed a Se-adequate diet (Renko et al., 2008). GPX4 and SELENOT are also indispensable for neural development in mice. GPX4 gene knockout induces embryonic lethality, and mice with conditional GPX4 knockout exhibit massive neurodegeneration before weaning (Seiler et al., 2008), which might be associated with selective loss of parvalbumin (PV) interneurons in mouse hippocampal and cortical regions (Wirth et al., 2010). Although GPX1 gene knockout does not affect the normal development of mice, GPX1 overexpression improves the differentiation of mouse embryonic stem cells into central neural stem cells, especially dopaminergic neurons (Gardaneh et al., 2011; Abasi et al., 2012). SELENOT is the only ER-resident selenoprotein whose knockout leads to embryonic lethality in mice. Neuron-specific SELENOT-knockout mice have significant decreases in the hippocampal, cortical, and cerebellar volumes due to neuronal apoptosis in the brain at postnatal day 7 (P7), leading to abnormal brain function in adult mice, which suggests a neuroprotective function of SELENOT in brain development (Castex et al., 2016).

Furthermore, SELENOW protects neurons against oxidative stress injury during neuronal development. H_2_O_2_-induced cell apoptosis is significantly increased in SELENOW-knockout primary neurons derived from the embryonic cerebral cortex (Chung et al., 2009). Recent studies of SELENOI (ethanolamine phosphotransferase 1, EPT1) showed that EPT1 mutation leads to cerebellar and brain stem atrophy, which can induce sensorineural deafness, blindness, and seizures. These results confirm the indispensable role of EPT1 in myelination and neurodevelopment and in the maintenance of phospholipid homeostasis in humans (Horibata et al., 2018). Although nervous system-specific deletion TXNRD2 does not affect the normal development of mice, cerebellar hypoplasia in TXNRD1-knockout mice occurs due to decreased proliferation of granule cell precursors within the external granular layer (EGL), indicating that TXNRD1 affects neuronal precursor cells and participates in the regulation of neuronal development (Soerensen et al., 2008). Thyroid hormone influences brain development through regulation of neuron and glial cell differentiation and myelin and synapse formation. DIO2 in astrocytes mediates the transformation of T4 into active T3, and DIO3 in neurons degrades T3 and T4 to stabilize thyroid hormone homeostasis in the brain (Bernal, 2000). Although the development and maturation of PV neurons in the cortex depend on thyroid hormone, no significant changes in these cells were observed in DIO2-knockout mice, and the most significant change was sensorineural hearing loss caused by retarded cochlear development (Ng et al., 2004, 2009). DIO3-knockout mice also exhibit developmental disorders in retinal receptors (Ng et al., 2010), indicating that DIOs can significantly affect the development of the visual and auditory systems in the brain.

#### Ca^2+^ Homeostasis and ER Stress

Seven ER-resident selenoproteins are involved mainly in regulating calcium flux, protein folding, and redox balance in the ER. Other selenoproteins are also involved in these physiological processes; for example, SELENOP responds to ER stress in hepatocytes (Zhao et al., 2016), and SELENOW regulates Ca^2+^ channels in muscle cells (Yao et al., 2016). However, according to current reports, only five ER-resident selenoproteins (SELENOK, SELENOM, SELENOS, SELENOT, and DIO2) participate in the regulation of ER homeostasis in the brain or neural cells. Previous studies have shown that the SELENOS-mediated complex, which is composed of SELENOK, valosin-containing protein (VCP) (an important ATPase on the ER membrane), Derlin (a chaperone protein), and an E3 ubiquitin ligase, transports misfolded proteins to the ubiquitin-proteasome system for degradation (Ye et al., 2004; Lee et al., 2015), indicating that SELENOS and SELENOK play important roles in protein folding and in the ER-associated degradation (ERAD) pathway. Neuronal SELENOS expression increases with ER stress (Curran et al., 2005; Gao et al., 2006), and SELENOS gene knockout results in ER stress-mediated apoptosis (Rueli et al., 2017). To date, no evidence indicates that SELENOS is directly involved in Ca^2+^ regulation. However, studies have shown that SELENOK can interact with a palmitoyltransferase (DHHC6) to affect Ca^2+^ flux through regulation of inositol 1, 4, 5-trisphosphate receptor (IP3R) palmitoylation (Verma et al., 2011; Fredericks et al., 2014) and that SELENOK overexpression increases IP3R-mediated free Ca^2+^ levels in microglia (Meng et al., 2019). Furthermore, a cyclic adenosine monophosphate (cAMP)-mediated increase in Ca^2+^ levels in neuronal cells was shown to significantly improve SELENOT expression. SELENOT overexpression also affects the basal Ca^2+^ level in cells, indicating that SELENOT regulates intracellular Ca^2+^ homeostasis (Grumolato et al., 2008). A study by Jo et al. (2019) in cell and animal models showed that mutation of Ala92 in DIO2 to Thr increased ER stress in different brain regions in mice and that the unfolded protein response (UPR) and hypothyroidism were present (Jo et al., 2019). Neuronal SELENOM overexpression has been shown to reduce H_2_O_2_-mediated intracellular Ca^2+^ flux. Conversely, SELENOM gene knockout promoted an increase in cytosolic Ca^2+^ levels, enhanced oxidative stress, and apoptosis (Reeves et al., 2010). In presenilin 2 (PS2)-overexpressing neurons, Ca^2+^ efflux from the ER was associated with a decrease in SELENOM expression (Hwang et al., 2005). However, the mechanism underlying the regulation of Ca^2+^ homeostasis by SELENOM remains unclear.

#### Synaptic Function and Neurotransmission

Because selenoproteins play diverse roles in development and maintain homeostasis in the CNS (Solovyev, 2015) selenoproteins may be assumed to play roles in signal transmission. Previous studies have shown that Se-mediated neurotransmission is active mainly in the dopaminergic system and that Se deficiency can induce chemical injury to dopaminergic terminals and neurons (Kim et al., 1999, 2000). However, few studies have confirmed the direct relationship between selenoproteins and neuronal signal transmission. SELENOP was the first selenoprotein identified to be associated with synaptic signal transmission. Alterations of synaptic transmission and long-term potentiation were observed in CA1 synapses of Selenop^–/–^ mice (Peters et al., 2006). In addition, SELENOP interacts with the postsynaptic apolipoprotein E receptor-2 (ApoER2) which participates in reelin protein-mediated synaptic signal transmission (Weeber et al., 2002; Beffert et al., 2005). ApoER2 also forms a functional complex with the N-methyl-D-aspartate receptor (NMDAR) and localizes in the postsynaptic membrane of excitatory synapses (Krebs et al., 1991; Beffert et al., 2005). NMDAR is the major receptor for glutamate during neuronal synaptic transmission, and synaptic impairment is directly associated with NMDAR disorders in AD (Wang and Reddy, 2017). Notably, a recent study by Zhang et al. (2020) showed imbalanced levels of two functional subunits of NMDAR, namely, NMDAR2A and NMDAR2B, in the brains of SELENOK-knockout mice. Therefore, SELENOK may play a role in neuronal synaptic transmission, but further studies are needed to support this speculation. Furthermore, neuron-specific GPX4-knockout mice and Selenop^–/–^ mice have spontaneous seizures since the corresponding regions (the cortical region in GPX4-knockout mice and the inferior colliculus in Selenop^–/–^ mice) in the brain lack PV-expressing GABAergic interneurons (Wirth et al., 2010; Pitts et al., 2012, 2015), suggesting that GPX4 and SELENOP participate in the process of GABAergic neuron transduction. Although SELENOR deficiency does not interfere with CNS development, the significantly downregulated expression levels of synaptic proteins and the synaptic receptor NMDAR can substantially affect the persistence of long-term potentiation and long-term depression (LTP/LTD) in the hippocampal CA1 region of the mouse brain (Shi et al., 2019). A study by Boukhzar et al. (2016) confirmed that SELENOT was necessary for dopamine production by dopaminergic neurons in PD model mice. During oxidative stress induced by neurotoxins, SELENOT regulates tyrosine hydrolase activity to increase dopamine levels, thus maintaining the functionality of dopaminergic neurons (Boukhzar et al., 2016).

#### Glial Cell-Mediated Neuroinflammation

Neuroinflammation in the brain is the excessive reaction of glial cells to pathological changes and usually presents as excessive activation and proliferation of microglia and astrocytes. The antioxidant capacity of GPX1 may participate in the regulation of the inflammatory cascade reaction in the brain. Gpx1^–/–^ astrocytes reduce the H_2_O_2_ clearance rate to cause cell death (Liddell et al., 2006; Shin et al., 2018). However, mice with ischemic brain diseases and GPX1 overexpression have significantly fewer overactivated astrocytes and microglia than corresponding mice without GPX1 overexpression (Ishibashi et al., 2002). Functionally, GPX4 is the only GPX that can use phospholipid hydroperoxides as substrates (Brigelius-Flohe and Maiorino, 2013) and is the control center for lipid oxidation-mediated apoptosis. In a neuron-specific GPX4-knockout mouse model, stress-induced proliferation of astrocytes and neuroinflammation associated with neurodegenerative diseases occurred in the brain (Seiler et al., 2008). Interestingly, compared to GPX4 expression during perinatal brain development, it decreased in glial cells in the adult human brain; however, GPX4 expression in astrocytes was found to be significantly upregulated in a cerebral ischemia model (Savaskan et al., 2007). The same phenomenon was observed for SELENOS. Compared to its high expression in neurons, SELENOS expression is sparse in astrocytes. However, under pathological conditions or in the setting of brain injury, SELENOS expression increases significantly, especially in reactive astrocytes. A study by Fradejas et al. (2011) showed that SELENOS overexpression influenced astrocyte functions and reduced the secretion of proinflammatory cytokines to enhance resistance to ER stress and neuroinflammation. As mentioned in the previous sections, SELENOK promotes the migration and phagocytosis of microglia through the regulation of Ca^2+^ flux, and SELENOK is the only reported selenoprotein to have direct regulatory functions in microglia (Meng et al., 2019). SELENOM may reduce the oxidative stress level in cerebellar astrocytes, in addition to neurons, through the regulation of Ca^2+^ flux (Reeves et al., 2010). Furthermore, brains from SELENOP- and SELENOR-knockout mice exhibit obvious glial cell proliferation, suggesting that these two selenoproteins might also be involved in the regulation of neuroinflammation in the brain (Valentine et al., 2005).

### Brain Function-Associated Selenoproteins and AD

**FIGURE 2:**
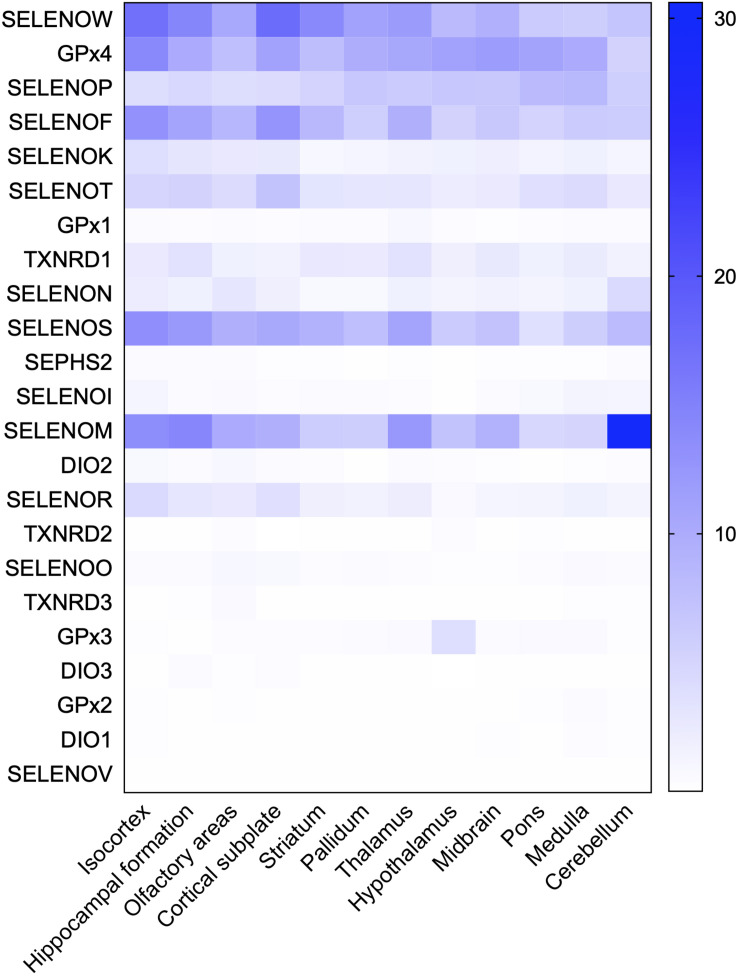
Heat map of the distribution of 24 selenoproteins in the mouse brain. Based on genome-wide *in situ* hybridization data published in the ABA for the brains of adult mice, raw expression values of 23 selenoproteins in 12 regions in the mouse brain were analyzed and used to plot a heat map. The database contains no expression data for SELENOH in brain regions.

The hippocampus, olfactory bulb, neocortex, and cerebellar cortex, which have high selenoprotein expression, are also vulnerable to neurodegenerative diseases; in particular, the hippocampus and cortex are major pathological regions in AD. A heat map of the expression levels of selenoproteins in 12 brain regions was plotted based on the *in situ* hybridization profile information in the ABA (Figure 2). This map shows that the high expression levels of the brain selenoproteins SEPHS2, SELENOK, SELENOR, DIO2, SELENOS, SELENOF, SELENOW, and SELENOT are even more pronounced in the hippocampal and cortical regions (the main pathological areas in the brain in AD) than in other brain regions, suggesting that these selenoproteins might be strongly associated with AD. Currently, research on selenoproteins in AD is limited. To further investigate the roles of selenoproteins in AD pathology, selenoproteins with the greatest research potential in AD are identified. Results in the literature were comprehensively assessed based on expression abundance in the brain, expression specificity in the hippocampal and cortical regions, and correlations of selenoproteins with four brain functions (closely associated with AD pathology). As shown in Table 1, selenoproteins were divided into 5 levels based on their expression levels (FPKM value: >100, 40–100, 15–40, 1–15, and <1) according to expression abundance in the brain. Selenoproteins were also divided into 5 levels based on their specific expression levels in the hippocampal cortex (percentage of expression in the hippocampal and cortical regions: >40, 30–40, 20–30, 10–20, and <10%), and divided into 2 levels based on the level of attention received in brain function studies (level 1: reported and level 2: massively reported), respectively. Based on the comprehensive coefficients (the total number of stars) obtained from the assessment, the top five selenoproteins are GPX4, SELENOP, SELENOK, SELENOT, and GPX1/SELENOM/SELENOS/SELENOW, which are consistent with the ranking of AD research correlation (the number of reports on each selenoprotein in AD-related research). Though this order may change with continued research on the roles of selenoproteins in brain function, these selenoproteins are hypothesized to be the most strongly associated with AD based on existing research data. Therefore, the relationships between these selenoproteins and AD are discussed further.

**TABLE 1:** Analysis of 25 selenoproteins in AD studies.

**Seleno-proteins**	**Expression abundance**	**Expression specificity**	**Brain function correlation**				**Comprehensive coefficient**	**AD research correlation**
			**Brain development**	**ER stress**	**Neuro-transmission**	**Neuro-inflammation**		
GPX4	✩✩✩✩✩	✩✩✩	✩✩	—	✩	✩✩	13	6
SELENOP	✩✩✩✩✩	✩✩	✩✩	—	✩✩	✩	12	9
SELENOK	✩✩✩✩	✩✩✩✩	—	✩	✩	✩	11	1
SELENOT	✩✩✩	✩✩✩	✩✩	✩	✩	—	10	0
GPX1	✩✩✩	✩✩	✩✩	—	—	✩✩	9	5
SELENOM	✩✩✩	✩✩✩	—	✩✩	—	✩	9	4
SELENOS	✩✩✩	✩✩✩	—	✩✩	—	✩	9	2
SELENOW	✩✩✩✩✩	✩✩✩	✩	—	—	—	9	1
SELENOR	✩✩	✩✩✩✩	—	—	✩	✩	8	2
DIO2	✩✩	✩✩✩	✩	✩	—	—	7	1
SELENOF	✩✩✩✩	✩✩✩	—	—	—	—	7	0
TXNRD1	✩✩✩	✩✩✩	✩	—	—	—	7	0
SELENOI	✩✩✩	✩✩✩	✩	—	—	—	7	0
DIO3	✩	✩✩✩✩✩	✩	—	—	—	7	0
SEPHS2	✩✩✩	✩✩✩✩	—	—	—	—	7	0
SELENON	✩✩✩	✩✩✩	—	—	—	—	6	0
TXNRD2	✩✩	✩✩	✩	—	—	—	5	0
SELENOO	✩✩	✩✩	—	—	—	—	4	0
TXNRD3	✩✩	✩✩	—	—	—	—	4	0
DIO1	✩	✩✩✩	—	—	—	—	4	0
SELENOH	✩✩✩	—	—	—	—	—	3	0
GPX2	✩	✩✩	—	—	—	—	3	0
SELENOV	✩	✩✩	—	—	—	—	3	0
GPX3	✩	✩	—	—	—	—	2	0
GPX6	—	—	—	—	—	—	0	0

*One star indicates 1 level, and the comprehensive coefficient is the total number of stars.*

#### GPX4 and GPX1

**FIGURE 3:**
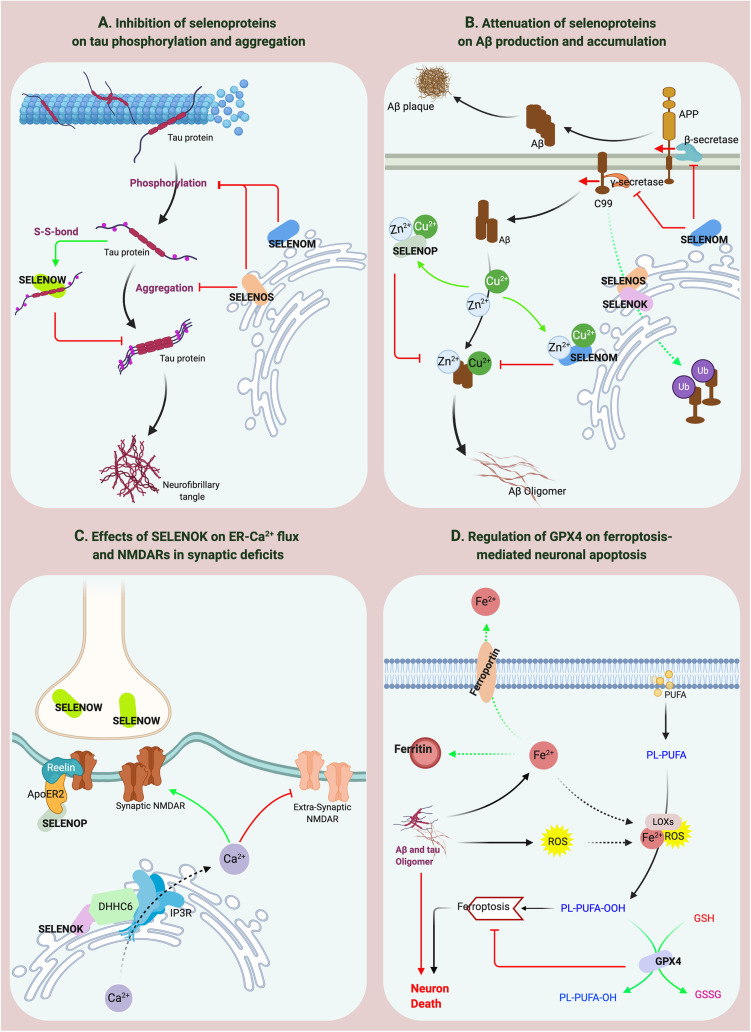
The effects of selenoproteins on the pathological processes of AD. **(A,B)** SELENOP, SELENOW and SELENOM inhibit the aggregation of Aβ and tau. SELENOS participates in the pathological protein degradation process through ERAD. **(C)** SELENOK affects the distribution of synaptic receptors. **(D)** GPX4 regulates ferroptosis-mediated neuronal apoptosis.

Ferroptosis is a current research hotspot. Enhanced neuroinflammation and elevated lipid oxidation levels are hallmarks of pathological changes related to ferroptosis in the brain (Hambright et al., 2017). Recent studies have shown that lipid peroxidation-induced ferroptosis participates in neuronal death in AD (Stockwell et al., 2017). GPX4 reduces lipid peroxide production catalyzed by Fe^2+^, and lipoxygenase (LOX) and is the key regulator of the ferroptosis pathway (Ingold et al., 2018; Wu et al., 2018). Significant iron accumulation and lipid peroxidation combined with reductions in glutathione (GSH) and GPX4 were observed in the hippocampus in AD patients (Yoo et al., 2010). In addition, GPX4 knockout in forebrain neurons of mice directly causes age-related neurodegenerative changes and obvious neuronal loss (Hambright et al., 2017), indicating that GPX4 is significantly associated with AD. Interestingly, the reactive oxygen species (ROS) level in GPX4-knockdown cells did not significantly change, but the level of lipid peroxidation products increased significantly. However, GPX1 does not decrease the lipid peroxide level mediated by GPX4 defects. These results suggest the specificity of GPX4 in protecting cells against lipid peroxidation damage (Yoo et al., 2010). Increased lipid peroxidation is considered an early event in AD. Iron accumulation promotes Aβ and tau aggregation (Yamamoto et al., 2002; Cheignon et al., 2018), whereas APP and tau collectively promote iron transport to cause the vicious cycle of ferroptosis (Duce et al., 2010; Lei et al., 2017). In the brains of Gpx4^±^ mice, the activity and protein level of β-secretase are significantly upregulated, and Aβ levels and amyloid plaque deposition are significantly increased (Chen et al., 2008). The above data indicate that the lipid peroxidation-mediated ferroptosis pathway regulated by GPX4 is involved in the neurodegenerative process in AD (Figure 3D), which may be a potential mechanism of Se on AD. Furthermore, as the downstream regulator of the ferroptosis pathway, the role of GPX4 in iron-mediated Aβ aggregation should be addressed, for the current data cannot confirm the direct effect of GPX4 on iron accumulation except for the inhibition of lipid peroxidation products.

Unlike for GPX4, the current research data on GPX1 cannot confirm its direct association with AD. Crack et al. (2006) used a GPX1-knockout primary neuron model and showed that depletion of GPX1 increased the sensitivity of neurons to Aβ toxicity. Similarly, the cognitive ability of GPX1-knockout mice treated with Aβ1-42 declined significantly, and the activity levels of the βII-isoform of protein kinase C (PKCβII) and extracellular signal−regulated kinase (ERK) in the brain significantly decreased. Re-expression of GPX1 in this mouse brain activated PKCβII-mediated ERK signal transduction to ameliorate Aβ1-42-induced memory impairment (Shin et al., 2020). In addition, studies of GPX4 and GPX1 gene polymorphisms show that certain GPX1 (rs1050450) and GPX4 (rs713041) genotypes are significantly associated with AD patients in a South Brazilian population (da Rocha et al., 2018). In addition, the Pro198Leu polymorphism in GPX1 has been reported to be associated with GPX1 enzyme activity. Recent studies found that this polymorphism can significantly affect the plasma Se level in AD patients, suggesting that the GPX1 genotype might impact the effect of Se supplementation in AD (Cardoso et al., 2012). Notably, in a survey of the Ecuadorian population, the Leu allele of GPX1 was a potential risk factor for AD (Paz-Y-Mino et al., 2010). However, no data are currently available to further validate whether the association between GPX1 polymorphisms and AD was due to the influence of GPX1 on Se levels in the body.

#### SELENOP and SELENOW

SELENOP is the most studied selenoprotein in AD-related research, possibly because of its high expression and multiple functions in the brain (Solovyev et al., 2018) and its association with the expression levels of many other selenoproteins. For example, loss of SELENOP can cause significant reductions in the brain expression levels of GPX4, SELENOK, SELENOM, and SELENOW (Hoffmann et al., 2007). Previous studies have reported that SELENOP expression increases in the brain with age (Lu et al., 2004) and that SELENOP gene expression is significantly increased in the brains of AD patients (Miller et al., 2008; Rueli et al., 2015). Bellinger et al. (2008) further confirmed that the spatial distributions of SELENOP, Aβ, and NFTs are colocalized in the brains of AD patients, suggesting that SELENOP is associated with AD. Ions of numerous metals, such as aluminum, zinc, copper, and iron, can promote Aβ aggregation during the pathological process of AD (Faller et al., 2014; Lei et al., 2020). Many Sec and Cys residues and a His-rich domain in SELENOP determine its metal-binding capacity (Turanov et al., 2015). SELENOP forms a coordination complex with Zn^2+^ and Aβ *in vitro* to inhibit Aβ aggregation and neurotoxicity (Du et al., 2013). SELENOP also interacts with the C-terminal domain of α-tubulin, which binds with tau protein to mediate the regulation of microtubule assembly (Du et al., 2014a), implying that SELENOP might be associated with the structure or function of tau protein. Studies by Du et al. (2014a) and Valentine et al. (2005) confirmed this hypothesis and showed that SELENOP significantly attenuates metalion-mediated tau aggregation and mitochondrial movement impairment and that SELENOP knockout induces structural and functional neuronal axon damage (Valentine et al., 2005; Du et al., 2014b). Furthermore, the antioxidant function of SELENOP also protects neurons against Aβ-induced toxicity. Interestingly, overexpression of Sec-deficient and histidine-rich SELENOP can inhibit the aggregation of Aβ and tau through the regulation of tropomyosin receptor kinase B (TrkB) signal transduction and Zn^2+^ homeostasis (Yue et al., 2020), significantly improving the learning and memory abilities of AD mice. These results indicate that the structural characteristics of SELENOP determine its potential active role in AD progression (Figure 3B). However, as Solovyev (2020) noted, although results clearly show that SELENOP is associated with AD, most relevant evidence is from *in vitro* studies. Therefore, more animal model and human-based studies are needed to obtain reliable conclusions (Solovyev, 2020). Moreover, compared with the inhibition of SELENOP on the aggregation of Aβ and tau, the interaction between SELENOP and ApoER2 and their effects on synaptic signal transmission deserve more attention as Figure 3C showed, which may be involved in synaptic dysfunction in AD.

Although dietary Se deficiency affects GPX activity in the brain, it does not reduce the SELENOW level (Sun et al., 2001). However, SELENOW in the brain is one of the selenoproteins whose expression is more affected by SELENOP (Hoffmann et al., 2007). Similar to most selenoproteins, SELENOW, as a GSH-dependent antioxidant, is involved in redox reactions (Jeong et al., 2002). SELENOW protects developing myoblasts against oxidative stress and inhibits interactions between 14-3-3 proteins and transcriptional activators to participate in muscle growth and differentiation (Loflin et al., 2006; Jeon et al., 2014). However, relatively few reports have addressed the role of SELENOW in brain function. SELENOW is highly expressed in the cerebral cortex, dentate gyrus, and hippocampus of postpartum rats and in the brain and spinal cord of developing embryos (Jeong et al., 2004; Chung et al., 2009). In addition, SELENOW is extensively expressed in synapses, and SELENOW expression is significantly decreased in the synaptosomes of SELENOP-knockout mice (Raman et al., 2013), suggesting that SELENOW has biological functions in neuronal synapses. A recent study by Chen et al. (2018) found that Cys37 of SELENOW and Cys322 of tau form a disulfide bond to inhibit tau protein aggregation, indicating that SELENOW may affect tau pathology and may be associated with AD (Figure 3A). However, the specificity of the disulfide bond between SELENOW and tau should be strictly evaluated. Based on the current data, the formation of a disulfide bond with tau at this site may be a general effect inhibiting tau protein aggregation, and further *in vivo* studies are needed to obtain more conclusive results.

#### SELENOK, SELENOT, SELENOS, and SELENOM

Our assessment indicates that ER-resident selenoproteins (SELENOK, SELENOT, SELENOM, and SELENOS), are strongly associated with AD, especially SELENOK, which has the strongest correlation due to its high expression in the brain and its specific expression in the cortex and hippocampus. Compared to other selenoproteins, SELENOK is highly expressed in immune cells (Verma et al., 2011). In addition, a Src-homology 3 (SH3) domain in SELENOK mediates its interaction with DHHC6, an enzyme that is also localized in the ER (Fredericks et al., 2014). The interaction between SELENOK and DHHC6 effectively catalyzes the palmitoylation of proteins such as IP3R and CD36 to stabilize their expression and further promote Ca^2+^ flux in the ER to activate immune cells (Fredericks et al., 2014; Fredericks and Hoffmann, 2015; Marciel and Hoffmann, 2019). Microglia-mediated neuroinflammation is considered a major AD-inducing factor (Tejera and Heneka, 2016; Hansen et al., 2018), and SELENOK overexpression significantly increases the Ca^2+^ level and IP3R expression and promotes the migration and phagocytosis of microglia to regulate neuroimmunity and neuroinflammation in the brain (Meng et al., 2019). However, the direct relationship between SELENOK and AD has not been reported until recently. A study by Zhang et al. (2020) showed that SELENOK expression is significantly decreased in the brains of AD patients and mice and that SELENOK knockout is associated with pathological changes, such as intracellular Ca^2+^ flux dysregulation in neurons and an imbalance in the distribution of synaptic receptors, that are highly consistent with AD pathology. Currently, neuronal excitotoxicity mediated by the disequilibrium between synaptic and extrasynaptic NMDAR is a widely accepted pathogenic factor for synaptic loss in AD (Hardingham and Bading, 2010; Talantova et al., 2013; Huang et al., 2017). As Figure 3C shows, SELENOK participates in the regulation of ER-Ca^2+^ flux and the balance between synaptic and extrasynaptic NMDAR expression to restore synaptic deficits in AD. In addition, the effects of SELENOK on immune regulation and microglia-mediated neuroinflammation in the brain should also be addressed, and further exploration of the underlying mechanisms may reveal the role of SELENOK in AD pathology.

Because of its effect on Ca^2+^ flux in neural cells and dopaminergic neurotransmission, SELENOT may be associated with AD. To date, however, no research has been conducted regarding SELENOT in AD. SELENOT expression is significantly increased in the peripheral blood mononuclear cells and brain striatal tissue of PD patients. Both silencing and overexpression of SELENOT influence oxidative stress and apoptosis in dopaminergic neurons (Boukhzar et al., 2016; Shao et al., 2019; Zhang et al., 2019). Dopaminergic neurons are also closely associated with AD pathology, and several alterations in the dopaminergic system have been reported in AD patients (Burns et al., 2005; Rossato et al., 2009). Dopaminergic neurons in the prefrontal cortex participate in the formation of cognitive memory (Perkovic et al., 2018). Loss of dopaminergic neurons affects synaptic plasticity in hippocampal CA1 neurons in AD (Nobili et al., 2017). Although the functions of dopaminergic neurons differ across brain regions, the regulatory function of SELENOT in dopaminergic neurons in AD-related brain regions warrants in-depth study.

Cleavage of APP by β-secretase under pathological conditions produces a 99-amino acid C-terminal transmembrane fragment of APP (C99), which is further cleaved into Aβ. Accumulating evidence indicates that a high C99 level is the determining factor of AD (Lee et al., 2006; Lauritzen et al., 2012; Pera et al., 2017). C99 is a miscleaved protein; therefore, the ERAD pathway is activated in cells for its degradation (Bustamante et al., 2013). As mentioned in the previous sections, binding of SELENOS to chaperone proteins such as SELENOK and Derlin can mediate the UPR and ERAD to maintain ER homeostasis. Recently, Jang et al. (2017) showed that ubiquitination-dependent C99 degradation was inhibited and that the Aβ1-42 level was significantly increased in a SELENOS-knockdown cell model of AD, indicating that SELENOS participates in the C99 degradation process through ERAD. Interestingly, there is no obvious relationship between the spatial localization of SELENOS and Aβ in the brains of AD patients. However, SELENOS is expressed at high levels in neurons of NFTs (Rueli et al., 2017). Further studies have shown that the inhibition of SELENOS expression under ER stress increases tau phosphorylation and phosphorylated tau aggregation. Although SELENOS is involved in the production of Aβ and the hyperphosphorylation and aggregation of tau protein (as shown in Figures 3A,B), the relationships between SELENOS expression and AD pathology proteins are still rather confusing, especially in mouse model studies. In addition, due to limited research on the effects of Se supplementation on SELENOS levels and enhanced SELENOS expression on cognitive ability, the mechanism of Se in AD from the perspective of SELENOS has yet to be clarified.

Previous studies have shown that the transcription of SELENOM is significantly inhibited in the brains of familial AD transgenic mice overexpressing a human mutant presenilin 2 (PS2) gene, which disrupt Ca^2+^ homeostasis through promoting Ca^2+^ shuttling from the ER to mitochondria (Hwang et al., 2005). Later, a study by Yim et al. (2009) confirmed that SELENOM participates in APP cleavage and tau hyperphosphorylation, that the activity of α/γ-secretases in SELENOM-overexpressing mice changed after Se treatment, and that the phosphorylation of tau protein at multiple sites was inhibited through ERK pathway activation. Unfortunately, these results have not been verified in AD models. Mutation of the Sec residue in SELENOM to Cys revealed that SELENOM can bind to transition metal ions via its His-rich domain and thus regulate Zn^2+^-mediated Aβ aggregation and neurotoxicity (Du et al., 2013). In an Aβ-expressing cell model, both full-length and truncated SELENOM were found to attenuate oxidative stress-induced mitochondrial damage through inhibition of Aβ oligomer formation (Chen et al., 2013). Still, the above anti-Aβ aggregation functions and mechanisms of SELENOM need to be further confirmed by *in vivo* studies, especially the effect and mechanism on homeostasis of Ca^2+^ and energy in the brain.

## Conclusion

As selenoproteins are the representatives of Se performing its physiological functions, investigation of the functions of selenoproteins in the brain and the association of selenoproteins with AD pathology might be critical for elucidating the mechanism of action of Se. Selenoproteins (GPX4, SELENOP, SELENOK, SELENOT, GPX1, SELENOM, SELENOS, and SELENOW), which are highly expressed in the brain, specifically expressed in AD pathological regions, and closely associated with brain function, may be the most promising targets in AD research. Existing reports show that these selenoproteins may participate in pathological processes of AD, including neuronal apoptosis, pathological protein aggregation and clearance, synaptic dysfunction, and glial cell-mediated neuroinflammation (Figure 3). Although the regulatory functions and molecular mechanisms of the above selenoproteins require further validation and exploration, this review provides relatively sufficient and reliable research data and directions for future studies on Se and AD.

## Author Contributions

Z-HZ: makes substantial contributions to conception and design, and literature research and annalysis of data, and the manuscript preparation and editing. Z-HZ and G-LS: participate in revising the manuscript critically for important intellectual content, and give final approval of the version to be submitted and any revised version. Both authors contributed to the article and approved the submitted version.

## Conflict of Interest

The authors declare that the research was conducted in the absence of any commercial or financial relationships that could be construed as a potential conflict of interest.
